# Visualization and Communication of Pharmacometric Models With Berkeley Madonna

**DOI:** 10.1038/psp.2014.13

**Published:** 2014-05-28

**Authors:** A Krause, P J Lowe

**Affiliations:** 1 Actelion Pharmaceuticals Ltd, Department of Clinical Pharmacology, Allschwil, Switzerland; 2 Novartis Pharma AG, Advanced Quantitative Sciences, Basel, Switzerland

## Abstract

Population or other pharmacometric models are a useful means to describe, succinctly, the relationships between drug administration, exposure (concentration), and downstream changes in pharmacodynamic (PD) biomarkers and clinical endpoints, including the mixed effects of patient factors and random interpatient variation (fixed and random effects). However, showing a set of covariate equations to a drug development team is perhaps not the best way to get a message across. Visualization of the consequences of the knowledge encapsulated within the model is the key component. Yet in many instances, it can take hours, perhaps days, to collect ideas from teams, write scripts, and run simulations before presenting the results—by which time they have moved on. How much better, then, to seize the moment and work interactively to decide on a course of action, guided by the model. We exemplify here the visualization of pharmacometric models using the Berkeley Madonna software with a particular focus on interactive sessions. The examples are provided as Supplementary Material.

## Applications of visualization

It is advisable to start the analysis by scanning the data for patterns and potential errors.^1,2,3^ Data exploration generally forms the first step toward understanding the data. Visualization as a key technique was discovered shortly after computers became widespread.^4^ Those visual displays are largely static.

Once exploration of the data has revealed patterns to be captured by the model, candidate model classes are assessed for their suitability of capturing these features of the data. A model class denotes a set of equations with parameters of unknown values. The model class is implemented in a visualization tool that allows variation of the model parameters and visualization of the resulting model for the given set of parameters. Visualizing a model helps the modeling scientist to understand its characteristics and the influence of different parameter values on the shape of the outputs. Berkeley Madonna is an ideal tool for this purpose for its simplicity and ease of implementation of a model.

When it is established that the candidate model class can capture the essential features seen in the data, the parameters can be estimated, typically in successively refined iterations. For example, the modeler might start by developing a suitable pharmacokinetic (PK) model, followed by a PD model for placebo subjects and finally a full PKPD model for all data, active and placebo.^2,3^

With the final parameter estimates available, the model needs to be qualified with evidence that the data could have been generated by the particular model. Visualization techniques such as plots of observed vs. predicted data are employed.^5^ Generally, it is not sufficient to demonstrate that the model captures the average or typical profile: it should also capture the variability of the data such that future experimental scenarios can be simulated with said variability. Visual predictive checks show the variability of simulations from the model against the variability observed in the data.^6,7,8,9^ This technique is purely graphical; the judgment is based on visual inspection. The visual impression can vary depending on visualization parameters such as binning intervals and quantiles chosen for comparison. The technique has found its way into standard modeling software such as NONMEM^10^ and Monolix.^11^

A good model captures all relevant features of the data—and not more: making it as simple as possible, but not simpler (often credited to Einstein). From this stage onwards, all further information comes from the model and simulations thereof. Different doses and concentrations can be simulated along with the (model-predicted) PD responses and their variability.

At this point, the model can exert its full potential: clinical teams and decision makers will query the model to estimate and quantify results of different scenarios: what can be expected if we give a higher or lower dose, if we treat patients longer, or if we change the study inclusion criteria? How many subjects can be expected below or above a certain threshold? What is the risk of seeing any safety parameter below a critical threshold?

Many colleagues in clinical teams and project teams are not familiar with pharmacometric concepts, let alone techniques. Frequently, analyses are based on concentration–response or dose–response figures (or, worse, tables) with readouts taken at single time points (often with last observation carried forward!). There is thus, very often, a communication gap between research scientists, toxicologists, occasionally statisticians, translational and later development physicians vs. kinetically minded numerical data analysts, also known as pharmacometricians. The question, therefore, is how best to bridge this communication gap?

We believe the answer is in better model and data visualization.

### Key principles

The key principles of good visualizations were established by eminent researchers in the field, in particular the works by Tufte.^12,13,14^ These general principles apply directly to clinical data visualization. Some of the key principles are summarized as follows^15,16^:

- Provide a clear message.
- Restrict visualizations to a few different types: varying types of display asks for familiarization with each new type of graph, requiring more time to understand and interpret correctly.
- Show the quantity of interest: if the measure of interest is the placebo-corrected treatment effect, show it (not the separate effect of active and placebo treatment—this leaves it to the reader to interpret the difference).
- Colors should be used only if they help interpretation: use intuitive colors, if any (for example green for clinical response (green = good), yellow for stable disease and red for disease progression (red = bad) or blue for male and pink for female data.
- Use plotting symbols such that they help interpretation: if the categories have an order (e.g., dose groups), use symbols with an intuitive meaning or ordering (letters A, B, C; dash (two edges), triangle (three edges), square (four edges), etc., or even, if space permits, more than one character, e.g., the dose administered in milligrams).
- Use lines only for ordered data (time profiles, ordered categories) but not for data with no inherent order (sex, race, etc.).
- Auxiliary faint lines help reading values more accurately, e.g., faint gray horizontal and vertical lines going through the axis tick marks.
- Indicate thresholds of interest: for change from baseline, indicate the line of no change (y = 0) or a particular threshold of interest (e.g., 75% reduction from baseline).
- For a time axis,
  - Choose appropriate time intervals and axis tick marks, e.g., 24 h, weekly, 28 days or dosing intervals.
  - Indicate dosing events, if not too many (small symbols—preferably arrows—or faint vertical lines). Alternatively, indicate the duration of treatment with a bar.

For PK/PD visualizations in particular,

- For the modeling results to have an impact, it is vital to communicate efficiently the final model's features to the clinical team and the decision makers. By means of both static and interactive graphics, clinical scientists can be guided toward a more complete kinetic understanding of dose–concentration–time–response relationships.
- To enable understanding of the model components, show them individually: absorption, elimination, time around Cmax (PK), placebo response, circadian rhythm (PD), etc., are understood more easily if they are not overlaid by other components.
- Show the quantity of interest: not only estimated response and confidence intervals but e.g., the estimated percentage of patients crossing an efficacy or safety threshold as a function of time, drug concentration, or dose (or combination thereof).

### The impact of interactive visualization

Model visualization is, nowadays, an integral part of the model development process and its importance is well recognized.^5,17,18^ Previously, scenarios were shown and discussed that were prepared beforehand, limiting the discussion. Now computers are faster and software is available to move to the next level, *interactive* model visualization. Interactive visualization allows what-if questions to be answered on the spot, providing the results of interest (almost) in real time. Questions about the impact of different doses, regimen, patient characteristics, or inclusion criteria can be answered in a meeting, making the discussion substantially more effective and efficient. We have illustrated some of the principles outlined earlier with applications in Berkeley Madonna.

Bonate^19^ defines the steps for model development as problem analysis, experimental conduct, data collection and cleaning, model formulation, model fitting, model checking, validation, interpretation, and communication (with iterations). We have focused on the steps that help understanding and communicating the model, including the exploration of alternatives as well as interpretation and communication.

### Working with clinical teams

*What are typical questions asked by drug development teams?* A typical well-defined question is “what is the minimum concentration or dose required to reduce blood pressure by at least 20 mm Hg prior to dosing on day 3 of treatment in at least 80% of all patients treated?.” Alternatively, “what dose and/or regimen is required to prevent a flare in patients' disease symptoms in at least half the patients in the 6 months after start of treatment?” or “if we design a phase 3 study like this (…) what can we expect the results to look like? How certain will we be?”; “How many patients should we study and when should the measurements be taken to achieve a statistically significant outcome with at least 80% chance?.”

Ideally, it should be possible to answer questions like these in a meeting with the key thought leaders of the clinical or project team. With appropriate simulation tools, this is possible. We introduce here two examples to exemplify what's possible; both have been used with project teams. We then return to the foundations, building on the basics to enable tutees to implement such simulations for themselves.

**Figure 1:**
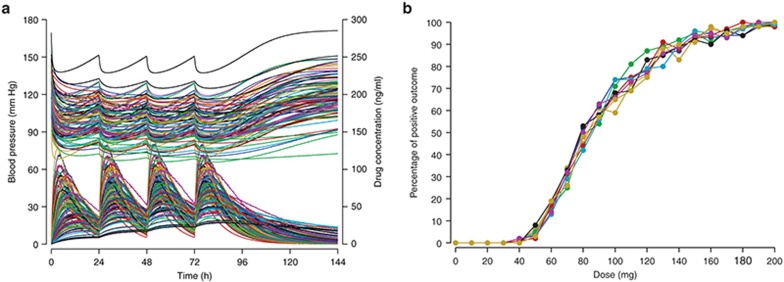
Clinical trial simulation for blood pressure change. Simulated drug concentrations and blood pressures (**a**, bottom and top curves) and fraction of patients experiencing a positive outcome (**b**, reduction of blood pressure by at least 20 mm Hg 72 h after start of treatment). Ten simulations are overlaid showing percentages of successful outcomes for doses from 0 to 200 mg. Colors indicate subjects (panel **a**) and simulated studies (panel **b**).

In **Figure 1**, 100 simulated concentration–time profiles are shown (left, bottom curves, and right-hand side axis) together with the corresponding simulated blood pressure measurements (top curves, left-hand side axis). The right-hand side figure has the statistical evaluation of 100 simulated patients for doses from 0 to 200 mg (in steps of 10 mg): the percentage of patients reaching a reduction from baseline of at least 20 mm Hg at 72 h after start of treatment. Ten such simulations were run and overlaid, illustrating the variability in the expected outcome. In this example, doses of 120 mg achieved the desired effect, on average (i.e., in about half the simulated studies), while doses of 150 mg achieved the desired effect in all 10 simulated studies—a more robust outcome and therefore better to test in reality.

**Figure 2:**
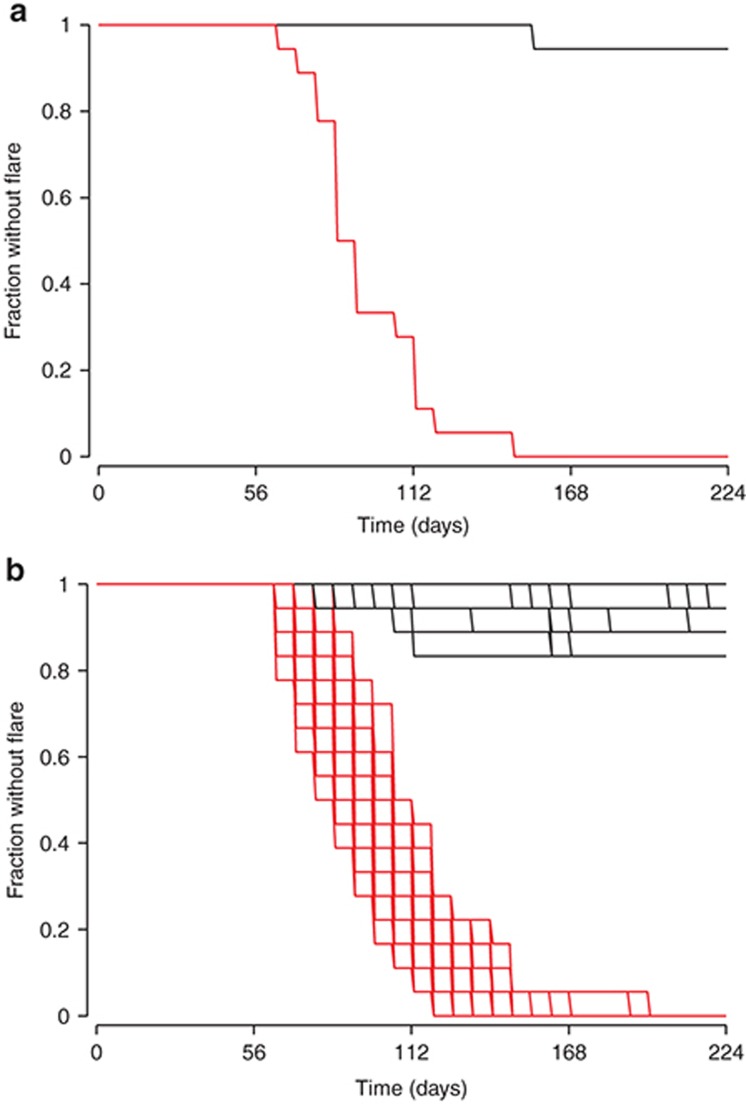
Fraction of patients experiencing no flares of inflammatory symptoms. Panel **a** shows a single trial simulation for 150 mg canakinumab, where 2 × 18 patients were treated from time zero, then randomized 1:1 to placebo at 56 days (red) or continued treatment (black). Panel **b** shows the simulation repeated 100 times.

**Figure 2** has a simulation of the proportion of patients exhibiting at least one flare or exacerbation episode of their symptoms, for a randomized treatment withdrawal after 56 days with 18 patients per arm. Because of the random selection of patients with different demographic characteristics and random variation in drug and system parameters, the simulated trial outcome varied from one simulation to the next. If the simulation was repeated 100 times and the results overlaid, the degree of variability one would expect to have in the trial outcome is visualized. The team can thus select the right dose and the right measurement times and minimize the likelihood of an inconclusive study. These simulations can be performed in a fraction of a second for a single trial or in about 10 s for 100 runs on a standard office computer, giving the team the chance to adjust the study design there and then. By the end of the meeting, the design is drafted and the team moves on to the practical work of setting up and running the study. This example is from the phase III planning for the anti-IL-1β monoclonal antibody, canakinumab (Ilaris) in cryopyrin-associated periodic syndromes. How was this done?

The model comprised the pharmacokinetics of the drug linked to a PD model for the probability of a flare in inflammatory symptoms, with parameters estimated by nonlinear mixed effects modeling in NONMEM.^20^ The model was then translated from NONMEM^10^ to the Berkeley Madonna software^21^ to enable, using a binomial (event) function, the simulation, accumulation, and plotting of inflammatory flares.

In this tutorial, the reader will be taken through the process of building a model, using a strategy that was already known among the Romans: *Divide and conquer.* Models can be quite complex, and understanding the components first before putting together the full picture makes the task easier and allows appreciation of the contributions of the different model components.^2,3,22^ There is first a general description of how to get started with the software via the PK, followed by PD and the inclusion of interindividual variability in parameters. Arrays of equations will be introduced, in order to simulate, simultaneously, several dosing arms of a study, for example. The arrays can be up to 3-dimensional, thereby allowing multiple dose levels, many individual patients, plus other key factors, as required. At the end of the tutorial, the introductory examples are reintroduced. In the end, tutees will be able to run and use the examples to create their own interactive simulations.

## Pharmacokinetics

Berkeley Madonna comes with a fast ordinary differential equation (ODE) solver and a visualization interface. The graphical user interface facilitates studying the effect of setting parameters to different values. The key components are the model visualizer, typically showing particular effects (e.g., drug concentration or a PD effect) over time, a slider to modify model parameter values by using the mouse, and an ODE solver that remains largely invisible. The system is arguably far from perfect, but the simplicity is part of the appeal. Models have to be coded in the Berkeley Madonna language. The key component, the differential equation system, is similar to other model specification languages such as NMTRAN (NONMEM), Monolix,^11^ or other systems.

On startup, Berkeley Madonna displays just a text editor with four lines, inserted by default (unless the options are customized):

METHOD RK4

STARTTIME = 0

STOPTIME=10

DT = 0.02

These lines specify the integration method (Runge–Kutta 4), the start time and the stop time of the integration interval, and DT or Δtime (delta time), the time intervals to be used in the numerical solving of the differential equation system. We recommend users to set the starting time to be before the first dose to prevent issues with calculating dose inputs (more on this topic follows later). Negative times are allowed.

A PK compartment can be defined by initializing it and then defining a differential equation with inputs (positive terms) and losses (negatives). The differential equation can be written as d/dt(comp1) as in the example above, or more succinctly using “prime” notation, i.e., comp1'. The amount of drug present in compartment 1 (comp1) at time STARTTIME is defined as 0. Negative times can be used, unlike in software such as NONMEM version 7.2 and earlier, which can facilitate better displays for PD when there are baseline or screening data. Then, a dose of 100 units is added into that compartment every 24 h, starting at time 0.

init comp1 = 0

d/dt(comp1)= pulse(100, 0, 24 ) ; 100 mg from 0 h every 24 h

There are several syntax options for defining differential equations. The following statements are equivalent (excerpt from the help file):

x' = expression

d/dt(x) = expression

FLOW x = expression

x(t) = x(t - dt) + (expression) * dt

x = x + dt * (expression)

The pulse function adds the specified amount of drug almost instantaneously, thus mimicking an injection. However, please note that it specifies the input as an isosceles triangle with a base of two integration steps (2*DT or DTMAX).

Clicking the “Run” button or pressing Ctrl-R will cause Berkeley Madonna to solve this differential equation system and open a graphics window that shows the amount of drug in compartment 1 over time. At this point in time, one should change STOPTIME to 100 (hours) to be able to see the 24-h dosing interval (the default is 10 time units, h here, thus showing a single dose only). Pressing Ctrl-R or clicking “Run” once more reruns the differential equation solver and displays the new result. An analogous example is shown in the first **Supplementary Video S1** describing a one-compartment central administration PK model.^23^

To mimic an oral drug, a second compartment is added and initialized with 0. An absorption rate constant, ka, and an elimination rate constant, ke, are defined; these move the drug around the system: absorption is defined as a transfer from comp1 (subtraction from the depot) to comp2 (addition to the central compartment or blood), and elimination as disappearance from comp2. We use variable names such as ka in computer code to denote the parameter k_a_.

ka=0.1 ; definition of absorption rate ; constant

ke=0.2  ;  definition of elimination rate ;  constant

init comp1=0

init comp2=0 ; compartment 2 (central compartment)

d/dt(comp1)= pulse(100, 0, 24) - ka*comp1

d/dt(comp2)= + ka*comp1 - ke*comp2

**Figure 3:**
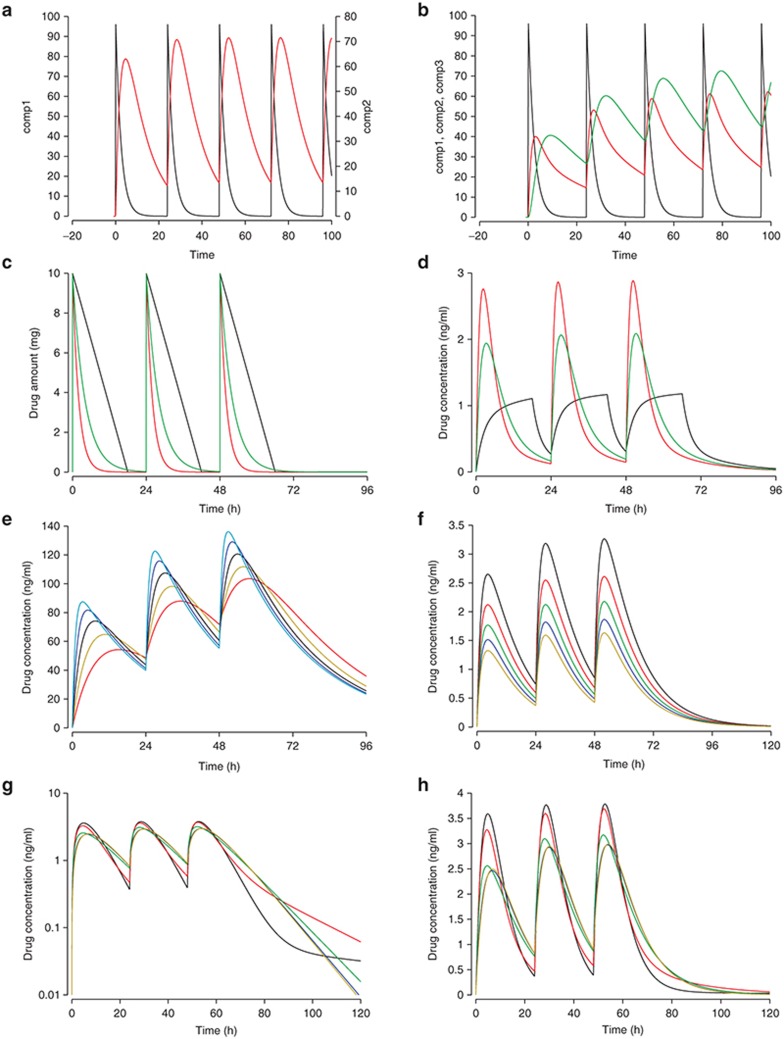
Visualization of parameter sensitivity. (**a**) One-compartment first-order model code showing drug amounts in the depot (black) and in the central compartment (red). (**b**) Two-compartment model with depot (black), central (red), and peripheral (green) compartments. (**c,d**) Drug amount in the depot (**c**) and drug concentration in the central compartment (**d**) for absorption processes of zero (green), first (black), and second (red) order. The effect of different (**e**) clearances (0.1, 0.2, … 1 liter/h, left) and (**f**) volumes (1, 2, 3, 4, 5 l) on drug concentrations. Effect of the intercompartmental transfer rate constant (0, 0.2, …, 1/h), (**g**) semilogarithmic and (**h**) linear.

Closing the graphics window and running the solver once more displays the amount of drug in both compartments (**Figure 3**). Alternatively, we could have double-clicked into the graphing area to select comp2 from the list of variables to be displayed on the “Y Axes.” Rerunning the solver is still necessary. Figures can be transferred to Office documents by simply using copy/paste then setting the background grey to be transparent. We will show later how to create publication quality graphical outputs.

We can now start modifying the display. Clicking on one of the buttons labeled “comp1” and “comp2” underneath the graph will make the corresponding compartment's curve appear and disappear. Holding the shift key and clicking will move the corresponding *y*-axis to the left or right, respectively. The two axes can have different ranges such that the visualization can change when variables are moved to another side.

Next, one can define a slider: a tool to modify, interactively, the parameter value. Clicking on Parameters-Define Sliders will open an interaction dialogue. Clicking on ka and then “>>Add>>” will add ka to the slider, similarly ke. Clicking “OK” will display the slider. Now, we can modify the parameters ka and ke interactively: clicking on the slider and moving it to the left and to the right will modify the parameter value and solve the differential equation system in (almost) no time.

Clicking the “O” button above the graph in the graphics window will cause all subsequent curves to overlay, thus displaying multiple absorption rates. Moving the slider and setting ka to 0.1, 0.11, … 0.2 yields a graph with the concentration–time profiles for the different absorption rate constants overlaid (for both compartments). Alternatively, instead of moving the slider manually, a batch run can be executed by selecting Parameters-Batch runs from the menu and specifying 11 runs of ka moving from 0.1 to 0.2. **Figure 3** shows examples of varying parameters and overlaying simulation results.

Adding a peripheral compartment and intercompartmental transfer rate constants, k23 and k32, is now straightforward. The code (header omitted) is as follows.

ka = 0.4; absorption rate constant (transfer ; from comp1 to comp2)

ke = 0.1; elimination rate constant (from ; comp2)

k23 = 0.3; compartment 2 to 3 transfer rate ; constant

k32=0.2; compartment 3 to 2 transfer rate ; constant

init comp1=0

init comp2=0

init comp3=0

d/dt(comp1)=-ka*comp1 +pulse(100, 0, 24)

d/dt(comp2)=+ka*comp1 –k23*comp2 +k32*comp3 -ke*comp2; central

d/dt(comp3) = +k23*comp2 –k32*comp3; peripheral

**Supplementary Videos S2 and S3** provide examples of both two-compartment distribution^24^ and first-order absorption.^25^

These are the basics of the Berkeley Madonna system. In the following, we are going to cover various aspects in more depth.

### Drug administration models

*Bolus.* Oral administration (tablet or capsule) is modeled as a bolus input into a compartment from which the drug is transferred into a central sampling compartment, e.g., the blood stream. A bolus is a direct addition of the drug into the central (blood) compartment.

The following code defines parameters for the amount of drug administered (dose), the dosing interval (dose_int), and the number of doses (ndoses). Furthermore, the PK parameters for absorption (ka) and elimination (ke) are parameterized.

METHOD RK4

STARTTIME=-1

STOPTIME=100

DT=0.02

; Parameter definitions

dose=100 ; dose amount 100 mg from 0 h onwards

dose_int=24 ; dosing interval

ndoses=3 ; number of doses

; PK parameters

ka=0.1 ; absorption rate constant

ke=0.1 ; elimination rate constant

; dosing input. Set to zero after ndoses

; administered

dosingperiod = if time < ndoses*dose_int-DT then 1 else 0

input = pulse(dose,0,dose_int)*dosingperiod

d/dt(comp1)=-ka*comp1 +input ; dosing compartment

d/dt(blood)=+ka*comp1 -ke*blood ; blood compartment

init comp1 = 0

init blood = 0

One may want to copy the code into an “Equation Window” in Berkeley Madonna and run it. Defining a slider allows modifying the parameter values with nearly instantaneous display of the result.

*Infusion.* Implementing an infusion model is relatively simple. The rate of change for the amount of drug entering the compartment is either equal to the infusion rate (during the infusion time) or zero outside the infusion time window. Dividing the current time by 24 and assessing if the remainder of the integer division is less than the infusion duration defines whether the current time is in the first 2 h (duration of the infusion) of the 24-h period (the dosing interval) our outside (when no infusion takes place). The modulo function provides the remainder of an integer division such that a 2-h infusion every 24 h with a rate of 7 units/h is coded as follows.

; infusion every 24 h for 2 h with rate 7

ke=1 ; elimination rate constant

init comp1=0

comp1_in=if (time>=0) and (mod(time, 24) < 2) then 7 else 0

d/dt(comp1)=comp1_in -ke*comp1

Berkeley Madonna's strength for visualization becomes apparent when the infusion model parameters are put on the slider: the user can change them easily by moving the slider with the mouse.

; infusion with parameters that can be put

; on the slider

ke=1       ; elimination rate constant

inf_rate=7    ; units/h

inf_duration=2 ; duration of infusion

inf_freq=24   ; infusion every X h

init comp1=0

; indicator for infusion ongoing: 1 (true)

; or 0 (false):

is_inftime=(time >= 0) and (mod(time, inf_freq) < inf_duration);

d/dt(comp1)=inf_rate*is_inftime -ke*comp1;

One can now visualize a two-compartment infusion model by putting the infusion parameters on a slider, using overlay mode (click “O” in the graph window), displaying the compartments' drug amounts by clicking the corresponding buttons underneath the graph window, and overlaying the profiles for 2-h infusions of 7 (units/time, e.g., mg/h) every 24 h with 1-h infusions of 7 mg/h every 12 h. You are encouraged to experiment: can you overlay oral administration and infusion and adjust the infusion rate such that the maximum concentrations in the central compartment are very similar? **Figure 3** shows some examples, including less common configurations such as a second-order absorption model.

### Absorption modeling

*Delayed absorption.* A delay in absorption of an oral drug, i.e., a time window between drug administration and start of absorption (during which the drug travels to the absorption site), is easily implemented by shifting the time interval for drug administration by the corresponding period of time. The code to be adapted is

tlag=0.5 ; absorption delay (lag) time

d/dt(comp1) = pulse(100, tlag, 24) -ka*comp1

*Absorption limitation.* If the drug amount that can be absorbed in a given period of time is limited (saturable process), the absorption can be capped at a value absmax and the absorption amount takes the value of absmax if the usual first-order absorption amount exceeds absmax: the absorption is defined as the minimum of the two quantities.

METHOD RK4

STARTTIME=-1

STOPTIME=100

DT=0.02

dose=100

ka=1

ke=0.05

absmax=20

abs_amount=min(absmax, ka*comp1)

init comp1=0

init comp2=0

d/dt(comp1)= pulse(dose, 0, 24) -abs_amount

d/dt(comp2)= -ke*comp2 +abs_amount

## PD Effects

### Circadian rhythm

**Figure 4:**
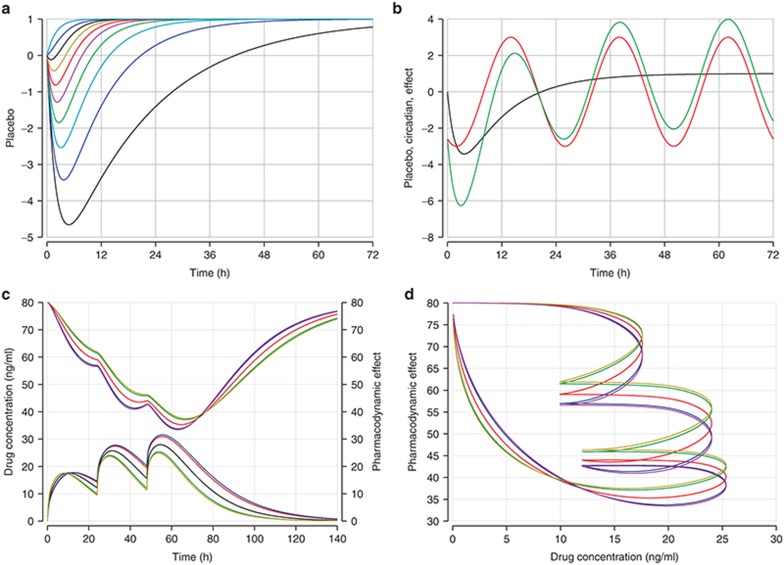
Pharmacodynamics. Placebo effects and circadian rhythms. Placebo effects with (**a**) different rates of increase and (**b**) placebo effects (black), circadian rhythm (red), and the combination of both effects (green). The indirect response model: (**c**) Pharmacodynamic effect (above) and concentration–time profile (below). (**d**) Time-delayed effect vs. drug concentration (hysteresis). Graphs **c** and **d** show a typical subject (middle line) and the 90 and 95% prediction intervals.

Circadian rhythms are commonly observed in PD responses. Consider, e.g., heart rate or total lymphocyte count, both of which change in a regular 24-h (*circa diem* = about a day) cycle. Some patterns are shown in **Figure 4**. A circadian pattern can be implemented by scaling the 24-h period length to the period length of a regular cosine wave, 2π:

amp = 1; amplitude, maxi change up and down

tshift = 10; time of maximum

circ = amp*cos(2*pi*(TIME-tshift)/24); circadian wave

Circadian rhythms can be additive but frequently are multiplicative; heart rate might vary by 10% during the day instead of by 10 beats/min. The PD effect is thus multiplied by the circadian component, (1+circ), where an amplitude of 0.1 denotes 10% variation during the day.

### Placebo effect

Treatment with a placebo can affect a measured response even though no active drug was administered; this can take many different shapes. A placebo effect can—with time—appear and disappear.

If the placebo effect appears at baseline and disappears gradually, a simple exponential form might suffice:

placebo_effect = exp(-exp(a)*time)

A more general placebo effect can gradually appear and only partially disappear. The following model defines the shapes that are shown in **Figure 4** (top left).

BL=100    ; baseline value

maxdown=0.1 ; max. decrease from baseline ; as fraction of BL

maxup=0.1 ; max. increase from baseline ; as fraction of BL

ratedown=1 ; rate of decrease

rateup=0.3 ; rate of increase

; derived parameters

down = maxdown*(1-exp(-ratedown*time))

up = maxup*(1-exp(-rateup*time))

placebo = 1+(up-down) ; placebo effect, multiplicative to Y

Y     = BL * placebo ; response, comb. of BL and plac. effect

### Response modeling

Functions provided by Berkeley Madonna can be called as in any other programming language. Writing a user-defined function, as one would in, e.g., R,^26^ is not possible. Currently, the only way to define one's own function is to write a “plug-in” function in C or C++, compile it, and then make the library file available for the software to call. The necessary documentation is available from the developers, but neither author has used this to date.

To characterize a PD effect as an *E*_max_ function of the drug concentration in the central compartment (direct effect), one would code

V2  = 33 ; Volume of distribution

C2  = A2/V2; concentration in compartment

E0  = 25 ; Base effect

Emax = 50 ; Maximum effect

EC50 = 40 ; Conc. required to reach Emax/2

PD  = E0 +Emax*C2/(C2+EC50); PD response

Similarly, a logistic response (typically a probability estimate) can be coded as PD = exp(*a* + b*slope)/(1 + exp(*a* + b*slope)).

An indirect effect requires an effect compartment with rates of appearance and disappearance. Note that there should be no drug transfer from a PK compartment to the PD compartment. Thus, adding a PD compartment will not change the code of the PK compartments and the code to be added is rather short:

; PD effect compartment

PD_in  = 0.1

PD_out  = 0.1

init PD = 0

d/dt(PD) = PD_in*C2 -PD_out*PD

**Figure 4** (row 2) illustrates a time-delayed effect and the associated variability (90 and 95% prediction intervals derived from 1.645 and 1.96 SDs around the (here single) random effect on absorption). The left side shows the PK profiles (below) and the associated PD response (above). The time shift (hysteresis) in the maximum concentration and the maximum effect is apparent. The right side shows evidence of hysteresis by plotting the PD effect (*y*) against drug concentration (*x*). Without hysteresis (i.e., an immediate effect), the graph would display a line moving from the left to the right: a particular drug concentration would always relate to the same PD effect. Since the PD effect (change) most often lags behind the driving force, the blood concentration (change), hysteresis polygons generally run counter-clockwise.

## Arrays

Berkeley Madonna allows the definition of arrays and, in particular, arrays of differential equations. This is a powerful tool that can be used for many purposes, including derivation and visualization of a larger number of differential equation systems.

### Transit compartments

The following code defines a transit compartment model,^19^ with a variable number of transit compartments that can be changed interactively by the user. The array structure allows for an elegant implementation of a transfer compartment model. The code below parameterizes the number of transfer compartments as ntransit. Note that this allows putting ntransit on a slider and modifying it interactively! You are encouraged to do just that now.

METHOD RK4

STARTTIME=-1

STOPTIME=5*24

DT=0.02

dose=100; dose amount, mg

ndoses=1; number of doses

dose_interval=24; dosing interval, h

ka=1; absorption rate constant, 1/h

ktr=0.25; transfer rate const btw transit; comp.

kout=0.25; transfer rate constant, 1/h

ntransit=15; number of transit compartments

init transit[1..ntransit]=0

init Aplasma=0

dosetime=if(time<ndoses*dose_interval-DT) then 1 else 0

; indicator for dosing period

d/dt(transit[1])=pulse(dose, 0, dose_interval)*dosetime

 -ktr*transit[1]

d/dt(transit[2..ntransit])= ktr*transit[i-1] -ktr*transit[i]

d/dt(Aplasma)=ktr*transit[ntransit]   -kout*Aplasma

**Figure 5:**
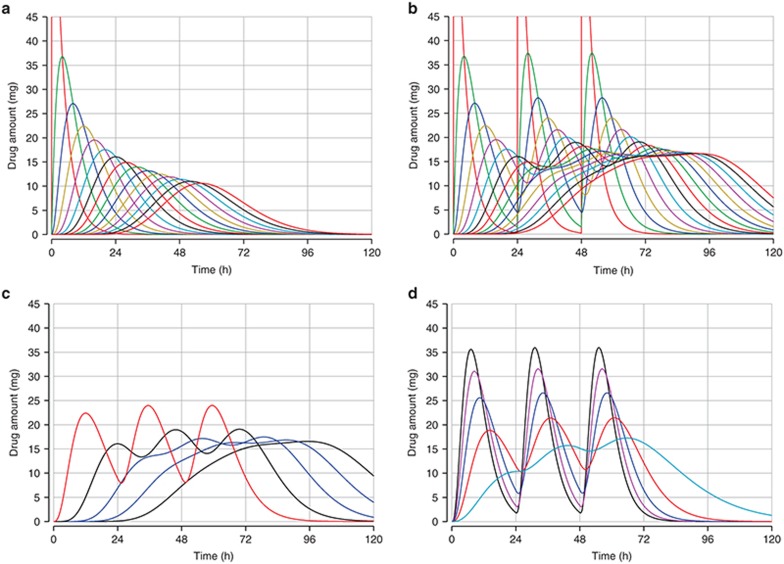
Visualization of transit compartments. Drug amounts present in each of 15 transit compartments with (**a**) a single dose and (**b**) three doses. Drug amounts in plasma with (**c**) 3, 6, 9, 12, and 15 transit compartments and (**d**) with transfer rates of 0.1, … 0.5/h for three transfer compartments and three doses.

**Figure 5** shows the drug amounts present in each of 15 transit compartments, the effect of the choice of the number of transit compartments on the drug amount in plasma, and the effect of different transfer rates for a given number of transfer compartments.

Arrays can have up to three dimensions. We will illustrate their use in the following sections.

### PK interacting with PD: target-mediated drug disposition

We have so far implicitly focused on modeling of molecular entities where the PD does not affect the PK. Some compounds, particularly drugs based on proteins but also some small molecules, can have the PK interacting with the PD due to quantitatively important amounts of drug binding to one or more targets. In this example, a drug is set to bind directly with a target according to a reaction,

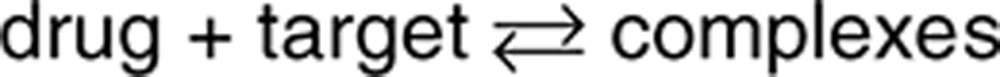


noting that while the drug enters the system by doses and is eliminated, the target (e.g., a receptor) is continuously supplied by the body and eliminated at a rate different from the drug. The complexes, formed by a reversible reaction, are also eliminated but at a rate different from that of the drug or the free target. The quantities of interest are the concentration of the free drug, CFD, and the total and free concentrations of the target, CTT and CFT. When running the code below in Berkeley Madonna, these quantities can be visualized over time. Note that the time unit here is days, based on a theoretical monoclonal antibody.^27,28^ The code also illustrates the use of arrays to display the PK and PD profiles for different doses. Since the differential equations are stiff, we use a particular differential equation solver, STIFF. We will describe the various types of differential equation solvers in more detail later. The DTOUT parameter allows the user to have displayed all integration time points (DTOUT = 0, the default) or only at selected time intervals.

METHOD STIFF

DTMIN = 1e-3

DTMAX = 0.1

DTOUT = 0

TOLERANCE = 1e-3

STARTTIME = -28

STOPTIME = 84

; All DE written in terms of molar mass of

; substance,

; not concentration, in order to maintain ; mass balance

; but note kon is second order with respect ; to concentration.

; Mass to moles and back requires molecular ; weight.

S'[1..n] = +input[i]-ka*S[i] ;injection site

D'[1..n] = -kon/V*D[i]*T[i] +koff*TD[i] -keD* D[i] +ka*S[i];drug

T'[1..n] = -kon/V*D[i]*T[i] +koff*TD[i] -keT *T[i] +RateT;target

TD'[1..n]= +kon/V*D[i]*T[i] -koff*TD[i] -keTD*TD[i];complexes

INIT S[1..n] = 0

INIT D[1..n] = 0

INIT T[1..n] = RateT/keT; initial, rate in ; divided by elimination

INIT TD[1..n]= 0

CTD[1..n] = 0.15*(D[i]+TD[i])/V

; µg/mLtotal, free plus complex

CTT[1..n] = 190 *(T[i]+TD[i])/V

; ng/mLtotal, free plus complex

CFT[1..n] = 190 * T[i]/V

; ng/mL free target

CFD[1..n] = 0.15* D[i]/V

; ng/mL free drug

DoseD[1..n] = 150*2^(i-2)

; mg doubling doses of antibody via array

DoseD[4..n] = 0

; extra arrays for color control

n=7

; use 7 arrays to rotate through 7 colors

tau = 28; days inter-dose interval

startt = 0 ; days start time

for dosing

kon = 1

; per Molar per time 2nd order rate const.

Kd = 1.5

; nM equilibrium dissociation constant

koff = Kd*kon; Kd=koff/kon so can

; reparameterise to kon&Kd

keD = 0.03

; (per d) could also be set as CL/V

keT = 0.5

; (per d) elimin. rate constant for target

keTD = 0.1

; (per d) elimin. rate constant for complexes

RateT = 50; (nmoles/d) input rate, or

; expression, of the target

V = 7; (L) volume in which binding

; reaction takes place

ka = 1 ; (per d) absorption rate

constant

input[1..n] = pulse(DoseD[i]/0.15,startt, tau)

**Figure 6:**
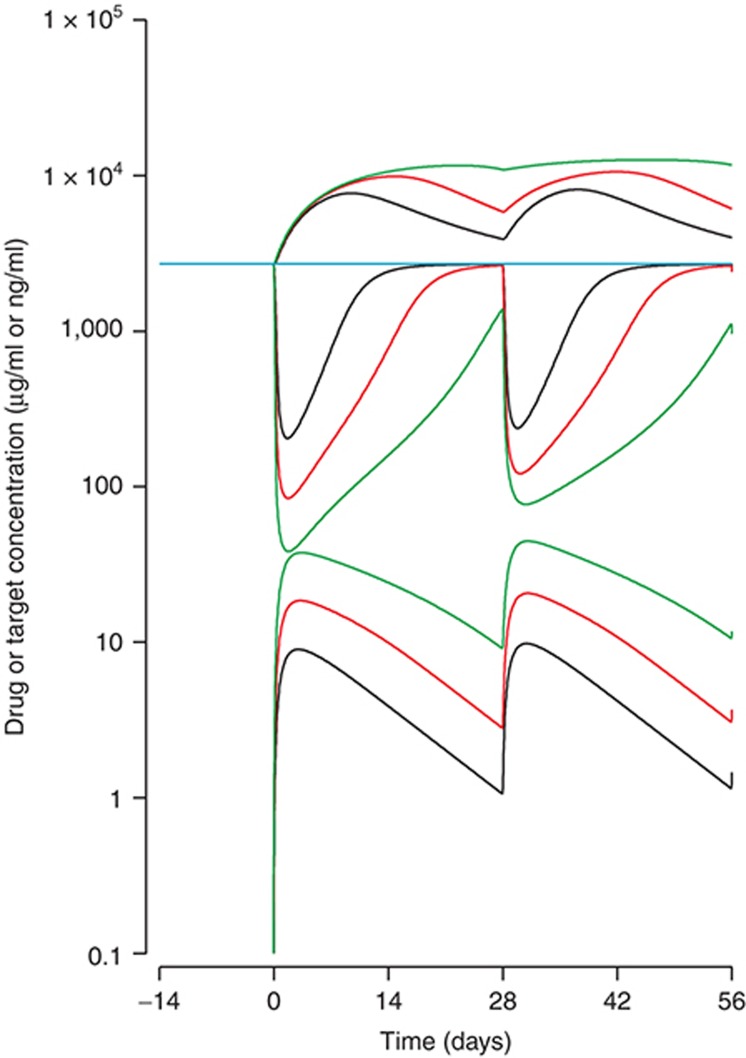
Visualization of target-mediated drug disposition. Total target (upper three curves), free target (center three curves), and free drug concentrations (lower three curves) on logarithmic scale. The colors indicate the doses of 75 (black), 150 (red), and 300 mg (green).

**Figure 6** shows that after doses of 75, 150, and 300 mg at 28-day intervals, the PK are distinctly nonlinear, exhibiting different profiles at low compared with higher doses. As the dose increases, the half-life appears to increase as the disposition of the drug changed from being controlled mainly by the elimination of the drug–target complexes, to being mainly the clearance of the free drug. Also, as the dose increases, the total target concentration increases, but only up to a limit—no more complexes could be formed than there is target available to capture. Given that the total target cannot increase indefinitely, the free target must therefore be suppressed in a dose-dependent manner according to the laws of mass balance (Le Chatelier's principle). Also noticeable is that the lowering of the free target concentration is greater on the first dose than the second and subsequent doses, due to the reversible formation and accumulation of drug–target complexes.

The next stage would be to quantitate links between either the formation of drug–target complexes or the suppression of free target, with clinical outcomes.

**Supplementary Video S4** provides an example for the binding model,^29^ describing how to set up equations for the second-order association reaction and first-order dissociation. The turnover components are not included, these being described above, but insight is given for how there can be differences in apparent experimental target or ligand binding (as described by an EC_50_, for example) vs. an underlying true *K*_D_ for the biochemical reaction. The situation is analogous to that observed in enzyme kinetics.

### Interindividual variation

Interactive Monte Carlo study or trial simulation, with parameters sampled randomly from distributions, is possible in Berkeley Madonna. However, only rudimentary tools are available, in particular the generation of uniformly and normally distributed random numbers. Multivariate normal distributions are not available directly but can be implemented.

The following code generates variability around population parameters and shows the results. The array conc contains the simulated concentrations to be visualized. Note also that we give one dose to the first subject, two to the second, and three to all others by putting the number of doses into a vector.

**Figure 7:**
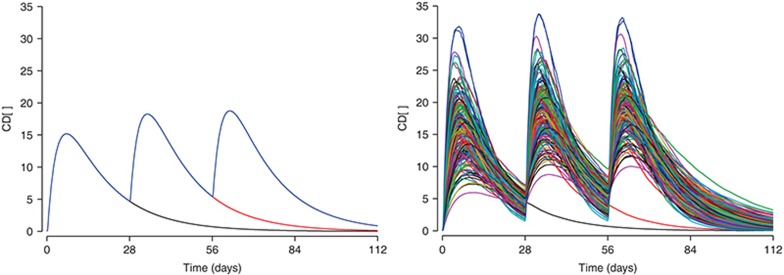
Simulation interactive. (**a**) One (black), two (red), and three doses (blue) administered to a population-typical subject. (**b**) Simulated profiles with variation in body weight and pharmacokinetic parameters CL, V2, and ka. Colors indicate subjects.

Note that we use a switch, MC, that is either 1 to switch on Monte Carlo generation of random numbers or 0 to switch it off. In practice, we always draw random numbers and perform the calculations on the full array, but if MC is set to 0, all subjects are identical to the population-typical subject. This method is obviously not efficient but generally feasible on a standard office computer. The resulting graphs for MC set to 0 and 1 are shown in **Figure 7**.

METHOD RK4

DT=0.2

STARTTIME = -1

STOPTIME = 4*28; days

MC = 1 ; Monte-Carlo switch (1=on) for

; variability

N = 200

; number of patients in each arm of trial

dose=1

idose[1..N] = dose; mg/kg

taudist = 28

tau[1..N] = taudist; days btw doses

Ndoses[1] = 1; one dose for first subject

Ndoses[2] = 2; two doses for second subject

Ndoses[3..N] = 3; all other subjects

BW = 70; body weight mean and std. deviation

BW_sdv = 10

; simulate body weights

init WT[1..N] = IF MC=1 THEN

  NORMAL(BW,BW_sdv) else BW

next WT[1..N] = WT[i];

; dosing

trtflag[1..N]=if (time<Ndoses[i]*tau[i]-DT)

then 1 else 0

input[1..N]=pulse(idose[i]*WT[i], DT,

tau[i]) * trtflag[i]

init S[1..N] = 0

init D[1..N] = 0

d/dt(S[1..N])= input[i] -ka[i]*S[i]

d/dt(D[1..N])= ka[i]*S[i]-D[i]*CL[i]/V2[i]

conc[1..N]=D[i]/V2[i]; display concentration

; population parameters and

; std. deviations (random effects)

CLpop = 0.2

CLsd = 0.2; standard deviation

V2pop = 3

V2sd = 0.3

kapop = 0.3

kasd = 0.3

; Generate random effects (uncorrelated)

init eta_CL[1..N]=NORMAL(0,1)*CLsd

init eta_V2[1..N]=NORMAL(0,1)*V2sd

init eta_ka[1..N]=NORMAL(0,1)*kasd

next eta_CL[1..N]=eta_CL[i];

next eta_V2[1..N]=eta_V2[i];

next eta_ka[1..N]=eta_ka[i];

CL[1..N]=CLpop*exp(eta_CL[i]*MC);L/d

V2[1..N]=V2pop*exp(eta_V2[i]*MC);L

ka[1..N]=kapop*exp(eta_ka[i]*MC);/d

The calculation time depends on the granularity of the evaluation of the ODE system and the number of calculations to be carried out. On a standard office computer, simulating up to 1,000 PK profiles interactively is feasible within just a few seconds. If the granularity is lowered by increasing DT, even more subjects can be simulated. The lower granularity may become visible as stair steps in the graph, however. The parameter DT can be put on a slider to study the effect of different values of DT.

### Statistical evaluation

Statistical evaluation of the simulations is possible, but only in rudimentary form. The user is largely restricted to calculating means and SDs of arrays. This enables one to calculate confidence intervals for arrays, similar to

m = arraymean(CD[*])

sd = arraystddev(CD[*])

int_high= m+1.96*sd

int_low = m-1.96*sd

The value 1.96 corresponds to the 97.5th percentile of the normal distribution such that the interval (q2.5%, q97.5%) yields the 95% confidence interval estimate. We used R to calculate the 97.5% quantile by entering qnorm(0.975).

Statistical tests or quantile functions to obtain the critical values for a statistic are not implemented. More sophisticated statistical evaluations would therefore require exporting the simulated data to a text file (e.g., csv) and evaluating it using another software system.

### Multivariate normal random numbers

A pharmacometric model frequently comprises a covariance matrix that has off-diagonal elements. Berkeley Madonna does not offer a routine to draw multivariate normal random numbers. However, it is possible, with some effort, to draw multidimensional normal random numbers.

Unfortunately, Berkeley Madonna requires SDs for the generation of normally distributed random numbers (in contrast to what the documentation states in versions up to 13 January 2010).^21^ The SDs have to be derived from the covariance matrix using the Cholesky decomposition. The decomposition derives a matrix *Z*, *Z*~*m x m*, from a given positive definite (covariance) matrix *y*, *y*~*m* x *m*, such that *Z*^*T*^*Z* = *y*. If a random variable *x* contains *m* independent normally distributed variables of length *n*, *x*~*n* x m, then *xZ* follows a multivariate normal distribution with covariance matrix *y*.^30^ Note that the Cholesky decomposition is particular for the given covariance matrix; if the matrix changes, the Cholesky decomposition and the random number generation must be updated manually.

For example, R can calculate the Cholesky decomposition by using the command chol.

# R code for Cholesky decomposition

COV <- matrix(

   c( 1.1,  0.6,  -0.1,

    0.6, 8.3,  -0.7,

    -0.1, -0.7,  4.0

    ),

    3, 3)

CH <- chol(COV); Cholesky decomposition, transposed

t(CH) %*% CH; yields COV matrix again

COV - t(CH) %*% CH; close to 0 (up to numerical inaccuracy)

Now, if we intend to generate three-dimensional correlated normal random numbers, the Cholesky decomposition from the matrix COV above is given as

 CH11 CH12 CH13 1.049 0.572   −0.095

CH = CH21 CH22 CH23 = 0.000 2.824  −0.229

 CH31 CH32 CH33 0.000 0.000 1.985

and the 3 one-dimensional vectors of standard normal random numbers are named eta1, eta2, and eta3, we can type the matrix multiplication into Berkeley Madonna:

N=47; number of subjects

rn=3; number of random effects

; random normal numbers N(0, 1)

init r[1..N, 1..rn]=normal(0, 1)

next r[1..N, 1..rn]=r[i, j]

CH[1,1]= 1.0488

CH[1,2]= 0

CH[1,3]= 0

CH[2,1]= 0.572

CH[2,2]= 2.824

CH[2,3]= 0

CH[3,1]=-0.095

CH[3,2]=-0.229

CH[3,3]= 1.985

; random effects, eta ~ MVN(0, COV)

eta1[1..N]=CH[1,1]*r[i,1] +CH[2,1]*r[i,2] +CH[3,1]*r[i,3]

eta2[1..N]=CH[1,2]*r[i,1] +CH[2,2]*r[i,2] +CH[3,2]*r[i,3]

eta3[1..N]=CH[1,3]*r[i,1] +CH[2,3]*r[i,2] +CH[3,3]*r[i,3]

The code above yields the three-dimensional normal random variate (eta1, eta2, eta3) with covariance matrix COV. The method is cumbersome, slow (calculations are carried out at each integration step!), and somewhat error-prone, but the authors do not know of a better alternative using Berkeley Madonna.

That the method works can be assessed within Berkeley Madonna by calculating the empirical covariance matrix of the random effects that should be close to the covariance matrix COV. The (*i*,*j*)^th^ element of the empirical covariance matrix is given as the average of the element-wise product of the random effects eta[i] and eta[j]:

; Testing for plausibility:

; Derivation of the empirical covariance

; matrix, Omega

Omega[1..rn, 1..rn]=0; Initialization

p11[1..N]=eta1[i]*eta1[i]

Omega[1,1]=arraymean(p11[*])

p22[1..N]=eta2[i]*eta2[i]

Omega[2,2]=arraymean(p22[*])

p33[1..N]=eta3[i]*eta3[i]

Omega[3,3]=arraymean(p33[*])

; Omega[i,j]=mean(eta[i] * eta[j])

p12[1..N]=eta1[i]*eta2[i]

Omega[1,2]=arraymean(p12[*])

p13[1..N]=eta1[i]*eta3[i]

Omega[1,3]=arraymean(p13[*])

p23[1..N]=eta2[i]*eta3[i]

Omega[2,3]=arraymean(p23[*])

; make Omega symmetric

Omega[2,1]=Omega[1,2]

Omega[3,1]=Omega[1,3]

Omega[3,2]=Omega[2,3]

Simulating a large number of subjects should yield an empirical covariance matrix close to the matrix specified. Looking at the numerical values by clicking the button 
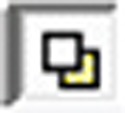
 in the visualization window will show mean covariances close to the original covariance matrix, COV.

### The blood pressure model

The Berkeley Madonna code for the blood pressure model introduced earlier is shown in its entirety below. It is also available for download online, complete with figures and sliders.

The code illustrates many of the aspects introduced in this paper, including PK and PD modeling, administration of a specified number of doses, simulation using random effects, the use of arrays, and evaluation of statistics (whether or not a particular clinical target is reached and how often it is reached in the simulated subjects at a specified time point of interest, here 72 h after start of treatment).

{

Simulation of PK (one compartment, oral absorption) and

PD (blood pressure) including random effects.

Clinical trial simulation can be conducted by using the

parameter plot and simulating doses, for example from

0 to 200 mg (show dose versus SuccessPercentT maximum).

Scenario:

Simulate nsim patients. Evaluate if success (reduction of

bp by at least 20 mmHg) is achieved at a specified

time point (e.g., 72 h after start of treatment).

}

METHOD RK4

STARTTIME = -1

STOPTIME=6*24

DT = 0.1

; Parameter setting

dose=130 ; dose amount (mg)

ndoses=4 ; number of doses to administer

nsim=100 ; number of patients to simulate

evaltime=3*24 ; evaluation at this many ; hours after start of treatment

PD_threshold=-20

; minimum change desired (success=PDchange ; < PD_threshold)

; PK parameters

CL=1

ka=0.1

ke=0.1

V1=1

; random effects (standard deviations)

eta_ka=1.0 ;

eta_V1=0.2 ;

eta_ke=0.01 ;

eta_BL=0.1; random variation at baseline

; (multiplicative)

; PD parameters

E0=125 ; baseline mean

Emax=-40

EC50=20 ; ng/mL

; Random effects

; individual random effects

; only generated at the start

; vector of individual ka values

init eta_ka_vec[1..nsim]= normal(0, eta_ka);

next eta_ka_vec[1..nsim]=eta_ka_vec[i];

ka_vec[1..nsim]=ka*exp(eta_ka_vec[i])

; vector of individual ke values

init eta_ke_vec[1..nsim]= normal(0, eta_ke);

next eta_ke_vec[1..nsim]=eta_ke_vec[i];

ke_vec[1..nsim]=ke*exp(eta_ke_vec[i])

; vector of individual volumes

init eta_V1_vec[1..nsim]= normal(0, eta_V1); mean, sd, seed

next eta_V1_vec[1..nsim]=eta_V1_vec[i]

V1_vec[1..nsim]=V1*exp(eta_V1_vec[i])

; PK

init depot[1..nsim]=0

drug2depot=pulse(dose, 0, 24) *

 (if (TIME < ndoses*24-DT) then 1 else 0)

depot2central[1..nsim]=ka_vec[i]*depot[i]

d/dt(depot[1..nsim])=drug2depot - depot2central[i]

init central[1..nsim]=0

d/dt(central[1..nsim])=depot2central[i] - ke*central[i]

; conc=amount/volume. error term added.

conc[1..nsim]=central[i] / V1_vec[i] * exp(normal(0, 0.01))

; PD

init BL_vec[1..nsim]=E0*exp(normal(0, eta_BL));

; add reff to baseline

next BL_vec[1..nsim]=BL_vec[i]

; PD effect

PD_change[1..nsim] = Emax * conc[i] / (conc[i] + EC50)

PD[1..nsim] = BL_vec[i] + PD_change[i]

; Treatment evaluation

; For the current time, assess for each ; patient if the desired effect

; is reached (declare success).

; NOTE: for increases desired, change less ; than to larger

; than,-Inf to +Inf in the constant below, and evaluate the

; min (not the max) of SuccessPercentT in ; the parameter plot.

Success[1..nsim] = PD_change[i] < PD_threshold

; Derive percentage of patients with ; success.

SuccessPercent = 100*arraymean(Success[*])

; If we are at the specified evaltime (or ; close to it),

; copy SuccessPercent into SuccessPercentT,

; otherwise set it to a very low number ; such that taking the maximum

; will not be affected by values not

; close to the time of evaluation.

; This is the quantity of interest.

SuccessPercentT = if (abs(time-evaltime) < DT) then

; SuccessPercent else -Inf

; The End.

### The PK-flare model

**Figure 8:**
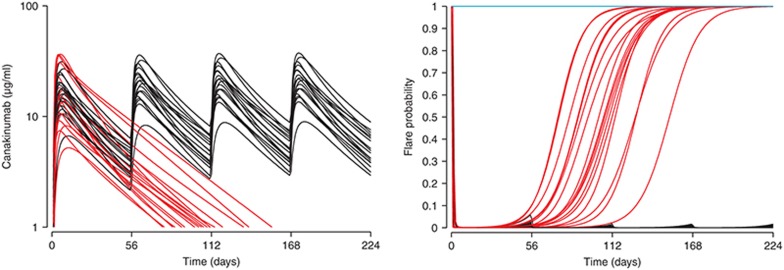
Canakinumab pharmacokinetic and probability of disease flare or exacerbation profiles. Both dosing arms received treatment up to 56 days in period 1. Thereafter, half were randomized to placebo (red) or continued treatment (black). The posology was 150 mg every 8 weeks or 2 mg/kg body weight if the body weight was <40 kg.

The code for the example outlined in the introductory **Figure 2** and the corresponding PK and flare probability profiles in **Figure 8** is given below, annotated to allow the reader to follow directly. The exacerbation or flare probabilities control binomial flare events which are accumulated to the Kaplan–Meier curves in **Figure 1**. Please note there are a number of tricks annotated within, e.g., to control the display of the seven available colors. The Berkeley Madonna file, complete with figures and sliders, is available online.

{

Canakinumab (ACZ885) PK flare model using

parameters as in Supplementary material

Table II of Lachmann JEM 2009; 206(5) 1029-36.

In vivo regulation of interleukin 1β in

patients with cryopyrin-associated periodic syndromes.

}

N = 18

; number of patients in each arm of trial

M = 7

; number of arms (only 2 used, but 7 to

; control colours across arrays)

Dose[1..N,1..2] = if WT[i,j]<40

 then 2*WT[i,j] else 150

; mg or 2 mg/kg for paediatrics <40 kg

Dose[1..N,3..M] = 0

; unused dose arrays

tau = 56

;days inter-dose interval

StartP2 = 56

; days, start time of study Part 2,

; initiation of counting of flares

STARTTIME= -28

; start time, best if before zero

STOPTIME = 224

; days to end of study Part 2,

; period where flare events are counted

NdosesP2[1..N,1] = 3

; number of doses in Part 2

NdosesP2[1..N,2..M] = 0

; number of doses in Part 2

visitfreq = 7 ;visit frequency in days

MC = 1 ;Monte-Carlo switch (1=on)

init WT[1..N,1..M] = IF MC=1

 THEN RANDOM(10,80) ELSE 70;kg body weight

next WT[1..N,1..M] = WT[i,j]

zz[1..o] = 1.1 ;dummy variable for

; controlling the 7 available colours. For

; example, if plotting 2 functions, use

; 5 dummies so colour returns to black

o=5

METHOD RK4

; integration method must be fixed

; step size for flare counting model

DT = 1; Integration time step

; (Euler, RK2, RK4 methods)

DTOUT = 0; Output time interval

; (0=store every step)

input[1..N,1..M] = PULSE(Dose[i,j]*F[i,j],

 DT,1000); first dose

input2[1..N,1..M]= IF TIME<=(2*DT+StartP2+

 Tau*(NdosesP2[i,j]-1)); Part 2&3 doses

 THEN PULSE(Dose[i,j]*F[i,j],StartP2,Tau)

 ELSE 0

S'[1..N,1..M] = input[i,j] +input2[i,j]

-ka[i,j]*S[i,j]; subcutaneous injection site

;central compartment

D'[1..N,1..M] = ka[i,j]*S[i,j]

 -D[i,j]*CL[i,j]/V2[i,j]

 +PS[i,j]*(T[i,j]/V3[i,j]-D[i,j]/V2[i,j])

;peripheral compartment

T'[1..N,1..M] = PS[i,j]*(D[i,j]/V2[i,j]

 -T[i,j]/V3[i,j])

init S[1..N,1..M] = 0

init D[1..N,1..M] = 0

init T[1..N,1..M] = 0

CTD[1..N,1..M]=D[i,j]/V2[i,j]

; concentration total drug

Pflare[1..N,1..M] = 1-CTD[i,j]^HILL/

 (KIEF[i,j]^HILL+CTD[i,j]^HILL)

;probability of flare

Visit = PULSE(DT,-28,visitfreq)

; returns 1 for a visit, else 0

Flare[1..N,1..M]=BINOMIAL(Pflare[i,j],1)*

 Visit; Flare event coinciding with a visit

Part3[1..N,1..M](t+dt) =

  IF TIME <= (DT+StartP2) THEN 0

  ELSE Part3[i,j] + Flare[i,j]

; count transfers to Part 3 during Part 2

init Part3[1..N,1..M] = 0

;initialise Part 3 counter

limit Part3 <= 1

;cannot transfer patient to

; Part 3 more than once!

Part3a[1..N]=Part3[i,1]

;separate first arm from 2D array

Part3b[1..N]=Part3[i,2]

;and second arm

Part3aSum = 1-ARRAYSUM(Part3a[*])/N

;sum up across N patients in first arm

Part3bSum = 1-ARRAYSUM(Part3b[*])/N

 ;sum up across N patients in second arm

Trial = 1

;counter for replicating the trials

; Parameters ACZ885 in Muckle-Wells

; disease, for 70 kg patients

CLmean[1..N,1..M]=0.181*(WT[i,j]/70)^1.0

; L/d

init CL[1..N,1..M]=CLmean[i,j]*

 exp(sCL[i,j]*MC) ;L/d

next CL[1..N,1..M]=CL[i,j]

V2mean[1..N,1..M] = 5.07*

 (WT[i,j]/70)^1.0; L

init V2[1..N,1..M]=V2mean[i,j]*

 exp(sV2[i,j]*MC); L

next V2[1..N,1..M]=V2[i,j]

{ variance-covariance matrix from Nonmem

0.0442 CL

0.0556 0.0869 V2

Choleski decomposition (from S-Plus) is

varmat1 <- matrix(c(0.0442, 0.0556,

0.0556, 0.0869), 2, 2)

dput(chol(varmat1))

structure(.Data = c(0.210237960416286, 0.,

 0.264462230749899, 0.130229522408659),

 .Dim = c(2, 2), .Dimnames=NULL, rank=2)

}

vA[1..N,1..M]=NORMAL(0,1)

vB[1..N,1..M]=NORMAL(0,1)

sCL[1..N,1..M] = 0.210*vA[i,j] + 0.0*vB[i,j]

sV2[1..N,1..M] = 0.264*vA[i,j] + 0.130*vB[i,j]

PSmean[1..N,1..M] = 0.103*(WT[i,j]/70)^0.667

;L/d

init PS[1..N,1..M]=PSmean[i,j]*

 exp(sPS[i,j]*MC) ;L/d

next PS[1..N,1..M]=PS[i,j]

V3mean[1..N,1..M] = 1.74*(WT[i,j]/70)^1.0

; L

init V3[1..N,1..M]=V3mean[i,j]*

 exp(sV3[i,j]*MC); L/d

next V3[1..N,1..M]=V3[i,j]

{ variance-covariance matrix from Nonmem

0.366 PS

0.0153 0.000651 V3

varmat1 <- matrix(c(0.366, 0.0153, 0.0153,

0.000651), 2, 2)

dput(chol(varmat1))

structure(.Data = c(0.604979338490167, 0.,

0.0252901198877037, 0.00337784488477104),

 .Dim = c(2, 2), .Dimnames=NULL, rank=2)

}

vC[1..N,1..M]=NORMAL(0,1)

vD[1..N,1..M]=NORMAL(0,1)

sPS[1..N,1..M]=0.605*vC[i,j]+0.0*vD[i,j]

sV3[1..N,1..M]=0.0253*vC[i,j]

 +0.00338*vD[i,j]

kamean=0.438 ; per day

ska=sqrt(0.185) ; stdev of ka

init ka[1..N,1..M]=kamean* exp(normal(0,ska*MC)); per day

next ka[1..N,1..M]=ka[i,j]

Fmean = 0.663

sF = sqrt(0.0881)

init F[1..N,1..M]=Fmean* exp(normal(0,sF*MC))

next F[1..N,1..M]=F[i,j]

KIEFmean = 1.13; µg/mL ACZ885 for

; 50:50 probability of flare

sKIEF = sqrt(1.58E-02)

init KIEF[1..N,1..M]=KIEFmean*

 exp(normal(0,sKIEF*MC))

next KIEF[1..N,1..M]=KIEF[i,j]

HILL = 4.22

;display CTD    ;Comment in or out to ;display function automatically.

;display Pflare    ;Or select manually.

display Part3aSum,  Part3bSum,  zz

## Model Fitting, Estimating Parameters

Berkeley Madonna allows simple least squares fitting of models to data. Data can be imported (Menu File-Import Dataset). The column indices for the *x*- and the *y*-variable must be specified. Selecting the menu (Parameters-Curve Fit) pops up a window that lets the user specify what parameters to use for fitting the model. Two guesses (original terminology of the input window) for the parameter values have to be given (why two remains unclear) and the fitting can start. It generally converges relatively quickly.

However, a least squares fit ignores the correlation structure in the data: it takes all observations as independent of one another, thus the association to the subjects is ignored. The parameter estimates can help, though, in finding the range of reasonable parameter values, e.g., to use them as starting values in NONMEM.

## ODE Solvers

A variety of algorithms are available for solving the differential equation system. They all have strengths and weaknesses.^31^ The Runge–Kutta 4 algorithm is robust for linear systems and is the default for a new project.

The Euler method is the oldest^32^ and numerically the easiest. The Runge–Kutta method (here with two and four stages) is quite fast and generally robust.^31^ The Rosenbrock algorithm^33^ serves better for stiff systems, but it can be quite slow and prone to error due to the need for very small step sizes (DT) at certain points in the integration. To quote Mathworks, “An ODE problem is stiff if the solution being sought is varying slowly, but there are nearby solutions that vary rapidly, so the numerical method must take small steps to obtain satisfactory results” (http://www.mathworks.com/company/newsletters/articles/stiff-differential-equations.html). The auto-stepsize method is an in-between solution for partially stiff systems. Note, however, that, as the name suggests, the stepsize varies with time between the limits DTMIN and DTMAX. This can lead to odd profiles around the time of dosing if the time points evaluated do not contain the exact time of dosing.

Increasing DT or DTMAX can substantially reduce calculation time, but at the expense of accuracy; the setting to be used is thus a compromise, but there must be sufficient accuracy to trust the results. The sensitivity of the results to the change can be visualized by putting DT on a slider and varying its value.

## Practical Tips and Tricks

Berkeley Madonna comes with comprehensive documentation. However, the material is concise and examples are kept to a minimum. This section provides practical tips for applications.

### Defining scenarios

Sets of parameters—model alternatives or scenarios—can be put on a slider, too. The idea is to move between scenarios by moving only a single slider that defines the scenario (and modifies a set of parameters). For example, consider the illustration of two competing models. Instead of having to modify the set of parameters one by one using the slider, one can modify the model number which in turn triggers setting all parameters to the corresponding values. Here is a simple example in which setting model to 1 or 2 changes the parameters ka and ke.

model=1; model selection switch

ka=if model=1 then 0.3 else 0.2

ke=if model=1 then 1 else 0.8

Alternatively, the parameters can be vectorized:

model=1; model selection switch

kavec [1]=0.1

kavec [2]=0.5

kavec [3]=1.0

kevec [1]=1

kevec [2]=2

kevec [3]=5

; set parameter values depending on the

; value of model (!)

ka=kavec[model]

ke=kevec[model]

This approach is helpful for comparing different scenarios and overlaying them. It has to be ensured when defining the slider that the parameter “model” cannot be set to an illegal, non-integer value or a value outside the range.

### Customizing graphs

Graphs in Berkeley Madonna can be customized. Double-clicking on a particular area such as an axis or the graphing area will pop up a window in which axis labels, tick marks, axis scales (logarithmic or linear), or transformations (Fourier) can be specified. The graph can also be zoomed into by selecting an area with the mouse. Un-zooming works like “undo”, clicking on the button labeled “Z” takes the zoom one step back at a time.

### Overlaying profiles

Different profiles can be overlaid. Clicking on the square button labeled “O” will keep the existing profiles and overlay them with the next profile selected. It is noteworthy that moving a slider to a new position will always create a new profile. If two parameters have to be changed to see another profile, it might be undesirable to see the profile after having changed the first parameter. An alternative is to open the parameter window (Parameters-Parameter Window), enter the values using the keyboard, and finally clicking “Run” in the parameters window.

### Parameter plots

Parameter plots allow studying the effect of a parameter systematically. For example, one can evaluate an effect (minimum, maximum, or average) for different doses. The function is accessible from the menu (Parameters-Parameter Plot).

The menu allows definition of the parameter to vary the variable(s) of interest and the statistic of interest (“Y Axes:”). For example, one can vary dose in 11 steps from 100 to 200 (obtaining the sequence 100, 110, 120, … 200) and show minimum, mean, and maximum concentration or PD effect. The result is a plot with dose on the *x*-axis and concentration on the *y*-axis, showing three lines (minimum, mean, and maximum concentration per dose). This feature allows relationships to be reviewed and the identification of thresholds, e.g., the largest dose such that the maximum effect is below a critical level.

In contrast to sliders, the parameter window is only updated after clicking the “Run” button, as repeated simulations are required.

Note that the statistics (min, max, mean) are calculated across all time points, which is may not be what is required. To calculate, e.g., the average trough concentrations for a set of doses at trough time (evaltime), 3*24 = 72 h, we calculate the statistic of interest, concstat, at each integration step, but we only store it into stats if we have arrived at the time of evaluation. Occasionally, the time of evaluation is missed by a tiny margin due to numerical inaccuracies such that we simply test if we have arrived in the vicinity of evaltime, not further away than a single integration step size, DT.

An example is given below. The statistics of interest, stats (equal to the average of the vector conc at time 72 h), can be displayed in a parameter plot against dose by showing min(stats) against a sequence of doses. Using the minimum is required since all values of stats that are not at the time of evaluation, 72 h, are set to a large value, Inf; averaging would yield nonmeaningful values.

Note that in order to obtain the maximum concentration at 72 h, stats must be set to a value that is certainly lower than the maximum concentration, e.g., -Inf, and the maximum of stats should be shown in the parameter plot.

ka=0.1

ke=0.2

dose=10

init depot=0

d/dt(depot)=pulse(dose, 0, 24) - ka*depot

init blood=0

d/dt(blood)=ka*depot - ke*blood

conc[1..100]=blood + normal(0, 0.1)

concstat = arraymean(conc[*])

evaltime=3*24; evaluation at this time

stats = if (abs(time-evaltime) < DT) then concstat else +Inf

### Seemingly inconsistent parameter values

It can be confusing at times to be clear about what parameter values the profiles shown correspond to. When the slider is moved, the curves are updated on releasing the mouse button. When values are entered into a parameter window, the profiles are updated after clicking on the “Run” button. The same occurs when using the parameter plots window. In such cases, parameter values and profiles shown are inconsistent.

In addition, there is the equation or code window that defines the parameters and their initial values. If a slider is present, the values on the slider are the current parameter values and the values in the code are ignored. If values are entered into the parameter window, the slider is updated directly.

Similarly, if parameters are modified in the parameters window but the “Run” button is not yet pressed, the visualization window is not updated and the currently shown model does not correspond to the currently displayed parameter values. Showing the parameters inside the graph by clicking on the square labeled “P” shows the parameters that were used for the current graph. The parameters window places an asterisk next to values that differ from the source code setting and these values can be reset by clicking on the “Reset” button in the parameters window.

### Initialization of arrays

Berkeley Madonna offers multidimensional arrays as data structure. They are defined by assigning values to the elements. Thus, assigning

x[1]=1

x[2]=2

creates a vector of length 2. Assigning x[1,1]=11, etc. creates a two-dimensional array or matrix. Initialization or declaration is therefore not necessary. The array expands in size as additional elements are defined. However, if an element is not assigned a value, e.g., if only x[1] and x[3] are assigned values, uncontrolled things can happen. To catch such accidental omissions, one can initialize an array with a large number, e.g., infinity, by coding x[1..n]=+Inf. The omission will become obvious in subsequent calculations since results will be implausible.

### Exporting Berkeley Madonna results for presentations and reports

At some point in time, the work must be documented—reverting to static visual displays. Graphics formats and options such as editing of graphs are fairly limited in Berkeley Madonna. The most striking “feature” is that all graphics windows have gray backgrounds. Using copy and paste to insert the graph into a slide set or a report will keep the gray background, making the graph hard to see.

Some office products allow pasting the graph into the document, double-clicking on it, and setting the “transparent color”: clicking on the gray background of a Berkeley Madonna graph will make it transparent. Whether setting the transparent color is possible depends on the graph format: e.g., bitmaps do not allow such modification. Using the “paste special” option instead of paste or Ctrl-V to insert the graph allows the user to select the particular graph format, e.g., Windows Metafile (called “Picture (enhanced metafile)” in current MS Office versions.

If multiple Berkeley Madonna graphs are created for the same presentation or report, it is advisable to set the size of the graph window in Berkeley Madonna to a particular size and never change it again. The font size is fixed and independent of the size of the window, and if the window is enlarged, the font size shrinks relative to the graph size. Thus, if graphs copied into a report originally had different sizes and are modified to have the same size, they will have different font sizes (for axis labels and tick marks, etc.).

It is possible to create publication quality figures directly from Berkeley Madonna. If one prints to a virtual printer such as Adobe Acrobat or PDFLite, a portable document format with vector graphics is produced. As suggested above, selection of an appropriate size of figure on the screen and suitable font size, together with printing to a smaller paper size, such as A5, A6, A7, or a custom size, is important for achieving a clear product.

For more freedom to adapt the graph to one's needs, it is common practice to export the table of data underlying a graph to a text file. In the Berkeley Madonna graph window, clicking on the icon shows two squares partially overlaid. The resulting table gives the data shown in the visual display. The menu “File-Save Table As” now allows storing the data to a text file. Reading the data into another system such as R adds more options for the creation of a graph. Note that scripting is more reproducible than mouse clicks.

### Licensing model

The Berkeley Madonna system offers a free version that is only slightly restricted in functionality: the code and the setup (screen layout) cannot be saved. The unrestricted version (version 8.3.18 as of this writing) can be purchased online and is priced quite reasonably. Version 9 is available as a beta version.^21^

Older versions of the software, such as version 8.0.1, are still in use at some institutions. The example code herein has been checked to ensure it runs in both 8.0.1 and 8.3.18, but the two have one distinct difference important to mention. The pulse function, very often used for drug input, has its isosceles triangle centered on the defined time in version 8.0.1. Therefore, if the start time is set to 0 and a pulse is administered at time 0, only half the quantity will enter the system. One should either delay the pulse by one integration step (DT or DTMAX depending upon the integrator), or set the start time to be before the first dose by at least one integrator time step. In version 8.3.18, the pulse has been redefined to be delayed by one integrator time step. If a defined finite number of doses is administered over a period of time, the modeler should check the last dose is administered correctly. It is possible, depending upon how the code is written, to experience half a pulse as the last dose.

### Berkeley Madonna sources

The Berkeley Madonna documentation is quite compact but contains basically everything needed. The help menu contains submenus “Equation help” and others that are well worth studying.

The only Berkeley Madonna user group of which the authors are aware was created on LinkedIn.^34^

One of us, Philip Lowe, created some years ago a series of short videos, each of 10–15 min.^23,24,25,29^ These demonstrate how to write and run models for the PK of a drug with one and two compartments, how to import and display data, simulate variability between individuals and a implement a drug-target binding model. The **Supplementary Videos S1–S4** are available online.

## Discussion

PKPD models can be and often are very complex. The modeler knows the model “inside out”, having spent time developing it. Now it is time for the modeling to make an impact: it must be communicated to the decision makers in the clinical team, pharmacologists, medical doctors, and managers.

If the model is not understood and only results are presented, the team might not believe the results—the background motivation is entirely lacking. Thus, the right balance between too little and too much detail is vital. The main components of the model must be motivated, frequently with a focus on the PD part.

As before, divide and conquer often proves a useful strategy. Illustrating the model components one by one, placebo effect, circadian rhythm, development of tolerance, etc., makes the model easier to understand and allows comparison with data (eye-balling is often sufficient to gain confidence). Once the components are understood, the full model can be presented. Limiting it to population-typical illustrations suffices, at least in the beginning. Varying parameters such as dose, body weight, or baseline PD will catch the attention and show how sensitive the PD response can be to these variations. Interactive displays are much more eye-catching than static slides.

Once the model is understood and confidence established, it is time to tackle the questions, as outlined in the introduction. This is frequently the time when we *move from modeling, as in creating the structure and estimating parameters, to simulation*. The distinction is not always clear to the non-pharmacometrics audience. The distinction must be made between

1. Simulation of a single large population to assess, e.g., the estimated percentage of subjects reaching a certain threshold in an efficacy or safety parameter. Estimation is based on counts: if 75 out of 1,000 simulated subjects reach the threshold, the estimate for the percentage reaching the threshold is 7.5%. Simulation for different doses will yield an estimated dose–response curve.
2. Repeated simulation of a study; e.g., 100 replicate simulations of a two-arm study with 40 subjects per arm. Such simulations allow for the estimation of uncertainties and power of a statistical test: how often was the difference between the two treatment arms statistically significant?

Simulation exercises can require more computing time than model parameter estimation, particularly if many hundreds of trials are simulated to gauge uncertainty in the predictions. Interactivity is thus only possible with reasonable computing time. The often used solution is to simulate anticipated scenarios and present static slides. However, in particular for exploration of scenarios with clinical teams, interactive simulation is highly desirable such that answers can be given almost instantaneously, keeping the discussion flowing.

Variations in the setting are important: they help understanding the sensitivity of the expected outcome as a dependency of the tuning parameters. With a better understanding of the relevance of the different parameters, the team can move toward decision making with better confidence. Interactive simulation helps since, if particular scenarios of interest are not covered, they can be simulated directly, avoiding the break in the discussion which would occur if the modeler had to go back to the office to produce the desired results.

## Conclusion

There is a fundamental difference between static and interactive graphics. Static graphics must be prepared before the discussion takes place, e.g., with the clinical or project team. Either the modeler prepares many visualizations, most of which will then not be used, or the risk is taken that a particular graph will not be available when the discussion requires it. Tools such as Pharsight's DMX (drug model explorer) help navigation through a large number of simulation scenarios prepared prior to the discussion.

The other possibility is the use of a tool that allows for visualization and even simulation interactively. Such tools include Berkeley Madonna and, recently, Monolix with its Model Visualizer MLXPlore.^35^ Both allow for interactive visualization by

1. Providing a suitable user interface, a slider that allows setting the parameters of the model to various values
2. Providing fast differential equation solvers, allowing for real-time calculation of even complex models.

Our experience shows that preparing all potentially interesting scenarios is unrealistic since too much computing and setup time is required, yet there is still a good chance that a scenario of interest is missed.

On the other hand, the creativity of team discussions is largely unlimited if a tool is available that shows the effects of the suggested scenario immediately, be it a different dosing regimen or different compound characteristics such as slow release formulations and the effect on the PD response.

Until this day, a modeling and simulation scientist requires a variety of tools: for data manipulation, data visualization, model fitting, qualification, visualization, simulation, and evaluation of simulated scenarios. A lean, integrated environment that allows for interactive, real-time visualization, simulation, and statistical evaluation comes closer to reality with computers becoming faster. Once such tools penetrate the pharmacometrics community, model development as well as visualization, scenario exploration and ultimately decision making will be raised to a new level.

## Conflict of Interest

The authors declared no conflict of interest.
